# Evidence for Prion-Like Mechanisms in Several Neurodegenerative Diseases: Potential Implications for Immunotherapy

**DOI:** 10.1155/2013/473706

**Published:** 2013-10-20

**Authors:** Kristen Marciniuk, Ryan Taschuk, Scott Napper

**Affiliations:** ^1^Vaccine and Infectious Disease Organization, University of Saskatchewan, Saskatoon, Canada S7N 5E3; ^2^Department of Biochemistry, University of Saskatchewan, Saskatoon, Canada S7N 5E5; ^3^School of Public Health, University of Saskatchewan, Saskatoon, Canada S7N 5E5

## Abstract

Transmissible spongiform encephalopathies (TSEs) are fatal, untreatable neurodegenerative diseases. While the impact of TSEs on human health is relatively minor, these diseases are having a major influence on how we view, and potentially treat, other more common neurodegenerative disorders. Until recently, TSEs encapsulated a distinct category of neurodegenerative disorder, exclusive in their defining characteristic of infectivity. It now appears that similar mechanisms of self-propagation may underlie other proteinopathies such as Alzheimer's disease, Parkinson's disease, Amyotrophic lateral sclerosis, and Huntington's disease. This link is of scientific interest and potential therapeutic importance as this route of self-propagation offers conceptual support and guidance for vaccine development efforts. Specifically, the existence of a pathological, self-promoting isoform offers a rational vaccine target. Here, we review the evidence of prion-like mechanisms within a number of common neurodegenerative disorders and speculate on potential implications and opportunities for vaccine development.

## 1. Introduction

Transmissible spongiform encephalopathies (TSEs), also referred to as prion diseases, are progressive, fatal neurodegenerative diseases characterized by neuronal loss, spongiform degeneration, and activation of astrocytes/microglia [1, 2]. Prion diseases have been defined in a number of species, which, despite sharing a conserved molecular mechanism, often display considerable inter- and intraspecies variability. Animal prion diseases include bovine spongiform encephalopathy (BSE) in cattle, scrapie in sheep, and chronic wasting disease (CWD) in elk and deer. Of the animal prion diseases only BSE is confirmed as zoonotic with demonstrated transmission to humans [3, 4]. Scrapie does not appear to be zoonotic and there is conflicting evidence on the ability of CWD to transmit to humans [5, 6]. 

 The threat of prion diseases to human health is quite low, although this is not always the case. Most infamously, during the 1950s outbreak of Kuru in the Fore tribes of Papua New Guinea, rates of human infection reached as high as 20% [7]. More recently, during the 1980s BSE outbreak in the United Kingdom, a novel form of CJD, emerged, infecting at least 227 people [8]. This new form of prion disease, termed “variant CJD” (vCJD), was linked to consumption of BSE-contaminated meat products. Outside these extraordinary circumstances, sporadic CJD (sCJD), which lacks an obvious genetic component, is the most common human prion disease [9]. It is unknown whether endogenous or exogenous factors contribute to sCJD [10]. Familial prion diseases account for about 5–15% of human TSEs and a number of mutations within the prion protein gene (PRNP) are disease associated [11–13]. These include Classic Creutzfeldt-Jakob disease (CJD), which occurs at a rate of one in a million people/year, Gerstmann-Sträussler-Scheinker disease (GSS) at a rate of five in 100 million people/year, and fatal familial insomnia, which has been characterized in 50 families [11, 14, 15].

## 2. A Novel Form of Infectivity

Prion diseases represent a novel paradigm of infection that is mediated by a protein agent, independent of agent-derived nucleic acid. This “protein-only” hypothesis revolutionized how we view and define infectivity. Infectivity resides in the misfolding of a normal cellular protein (PrP^C^) into a pathological and infectious conformation (PrP^Sc^). Propagation of prion infection, within and across animals, occurs through the ability of PrP^Sc^ to promote PrP^C^ misfolding in an autocatalytic process [16]. PrP^C^ is converted to PrP^Sc^ in a manner highly dependent upon species, prion strain, and genetic background [13, 17–21]. PrP^C^ is essential for infection and disease as PrP-deficient animals resist prion infection; restoration of PrP^C^ expression returns prion susceptibility [22]. Interestingly, in the same article where this novel mechanism of protein-based infectivity was first proposed, the authors hypothesize similar mechanisms of self-propagation in other protein misfolding diseases [16].

## 3. Mechanisms of Conversion

**Figure 1:**
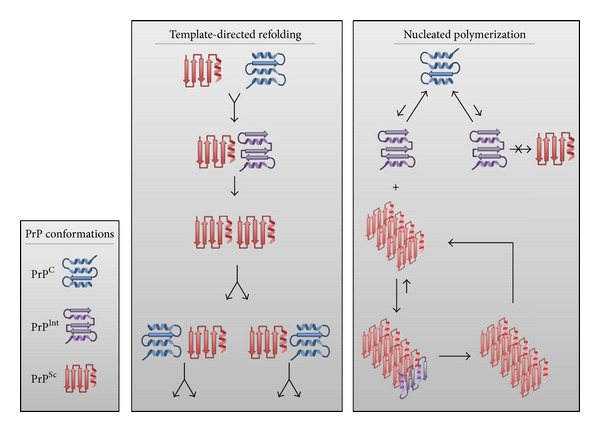
Proposed models of PrP^Sc^-induced misfolding of PrP^C^. The prion protein normally adopts a mainly alpha-helical structure under homeostatic cellular conditions (PrP^C^). PrP^C^ can potentially misfold to predominantly beta-sheet structure, thereby adopting an infectious and disease-causing conformation (PrP^Sc^). Many possible intermediate conformations of variable secondary structure, composition can be adopted during transition from PrP^C^ to PrP^Sc^ (PrP^Int^, denoted as a single structure for clarity).

There is considerable interest in defining the molecular mechanisms of PrP^Sc^-induced PrP^C^ misfolding, in particular if similar mechanisms are shared by other protein misfolding diseases. Two distinct models of conformational infectivity have emerged, template-directed refolding and nucleated polymerization (Figure 1). The template-directed model suggests PrP^Sc^ triggers a PrP^C^—fueled misfolding cascade in which PrP^C^ is a substrate for the reaction and newly generated PrP^Sc^ converts subsequent PrP^C^ molecules, thus propagating the cycle and amplifying the infectious material. In this context, PrP^Sc^ lowers the energy barrier that limits spontaneous conversion of PrP^C^ to PrP^Sc^ [23]. The nucleated polymerization model describes a thermodynamically controlled, noncatalytic, nucleated polymerization reaction in which conversion of PrP^C^ to PrP^Sc^ is a reversible process. PrP^C^ is highly favored at equilibrium and misfolding only occurs upon contact with a PrP^Sc^ aggregate. The PrP^Sc^ conformation is stabilized when newly misfolded protein is added to the aggregating seed. A primary consequence of this second model is that infectivity depends on the presence of PrP^Sc^ oligomers, as monomers are not infectious [23].

## 4. Additional Complexity in the PrP^C^/PrP^Sc^ Model

This basic model, in which PrP^C^ and PrP^Sc^ represent the healthy and abnormal forms of the protein, respectively, has been a valuable starting point to understand this unique mechanism of infectivity. This simple binary model is, however, insufficient to explain all aspects of prion disease. For example, while PrP^Sc^ is described as the infectious conformation, subtle variations exist that complicate the definition of the exact infectious component. For example, PrP^Sensitive^ (PrP^Sen^) and PrP^Resistant^ (PrP^Res^) differ in their sensitivities to Proteinase K (PK) digestion. While most PrP^Sc^-infected tissues contain PrP^Res^, this is not an absolute requirement of infectivity [24, 25]. PrP^Sen^ is also present in PrP^Sc^-infected tissue, complicating the assignment of infectivity to a specific conformation [26, 27]. There is also considerable evidence for the existence of multiple PrP^Sc^ isoforms, termed strains, with unique properties of infectivity, species tropisms, pathology, neurotropism, and biophysical traits [28]. Similar uncertainties are associated with the biological function(s) of PrP^C^ as well as the pathological mechanism(s) of PrP^Sc^. There appears to be an emerging consensus that PrP^C^ serves a neuroprotective function such that conversion of PrP^C^ to PrP^Sc^ may result in an undefined combination of a loss of this neuroprotective function of PrP^C^ or a gain in neurotoxic function of PrP^Sc^ [29].

 Appreciation of the complexities within the prion model may assist in understanding the mechanisms of self-propagation and pathologies, of other equally complex protein misfolding diseases. For example, the hallmark plaques of AD do not clearly correlate with dementia, challenging the assumption that these aggregates represent the primary pathological entity [30]. Such seeming inconsistencies highlight the need for better understanding of the agents and mechanisms associated with the proteinopathies. Specifically, that consideration of the proteinopathies from “folded-correctly versus folded-incorrectly” perspective likely oversimplifies the isoforms and pathogenic mechanisms. Critical aspects of disease may associate with subtle, low abundance isoforms and their aggregates. Further, the consequences of these conformational species may reflect undefined contributions of gain, loss, or change of function. 

## 5. Are Prions the Only “Infectious” Proteins?

There are a number of parallels between TSEs and other neurodegenerative diseases including Alzheimer's disease (AD), Parkinson's disease (PD), Amyotrophic lateral sclerosis (ALS), and Huntington's disease (HD). Most notably, these diseases all represent proteinopathies, defined by the misfolding of a self-protein into an aggregate structure. Outside the context of TSEs, the protein aggregates associated with these conditions are typically viewed as a consequence, rather than a cause, of disease. In recent years, however, there have been indications that Prusiner's prediction of prion-like mechanisms in a spectrum of protein-misfolding diseases may be quite prophetic. There is increasing evidence that mechanisms associated with prion self-propagation are conserved, to varying extents, in other proteinopathies. Exogenous amyloids of the various causative proteins of these diseases (A*β*42 and tau for AD, polyQ repeat expansions in Huntingtin, *α*-synuclein for PD, and SOD1 for ALS) induce misfolding of their naturally structured counterparts in cells, tissues, and animal models. Thus, misfolded protein aggregates are not only a pathological hallmark of these diseases, but also a key player in disease initiation and progression.

 Before beginning a detailed consideration of prion-like mechanisms within these diseases, it is appropriate to define and differentiate the terms infectious and self-propagating. Self-propagation describes mechanisms in which interaction between the natively folded and aggregated proteins induces misfolding of the natively structured protein. In contrast, infectious describes acquisition of an exogenous, disease-causing agent from an infected host or an environmental source. Prion diseases, with their well-documented transmission through animal populations, as well as zoonotic, iatrogenic, and cannibalistic transmission to humans, are clearly infectious. In contrast, it is unlikely, at least under normal circumstances, that AD, HD, PD, and ALS represent infectious diseases. There is, however, evidence that these diseases do self-propagate within an individual. Specifically, the misfolding proteins that serve as the basis for each disease share a common characteristic of being able to promote the misfolding of their properly folded counterparts. This mechanism appears to underlie, or at least contribute to, transmission of the misfolding events from cell-to-cell within tissues, between tissues, and throughout the host. A summary of the evidence implicating prion-like mechanisms within these diseases is presented in Table 1.

## 6. Prion-Like Mechanisms in Alzheimer's Disease

An estimated 36 million individuals suffer from Alzheimer's Disease worldwide [31]. The brains of AD patients are characteristically populated with plaques composed of A*β* peptide as well as neurofibrillary tangles of a hyperphosphorylated isoform of the tau protein [32]. While most of the current treatments for AD have prioritized the symptoms rather than the causes of AD, it is encouraging that a number of vaccine clinical trials are underway. Interpreting the outcomes of these trials, as well as strategies for future vaccine development, will likely be influenced by the appreciation and perspective of AD as a prion-like disease.

The first indication of a prion-like mechanism in AD came from the demonstration of A*β* plaque transmissibility in primates intracerebrally injected with human AD patient brain tissue [84, 85]. This phenomenon was later recapitulated through cerebral injections of brain extracts taken from AD patients into one side of the brain of transgenic mouse models of AD. The development of *β*-amyloid (A*β*) peptide plaques in these animals and the localization of plaques to the side of the brain receiving the injected material indicate that a component of the AD brain extracts, although not necessarily *β*-amyloid, initiates plaque formation [39]. Pretreatment of these AD brain extracts with antibodies to neutralize *β*-amyloid inhibited the ability of the extracts to initiate amyloid formation. This offers strong support that *β*-amyloid represents a toxic, self-propagating agent [42]. Similarly, stainless steel wires coated with AD brain extract caused *β*-amyloid plaque formation when implanted into the brains of mice. These deposits transmitted from the point of infection into neighboring regions of the brain [40]. The most striking similarity to prion infection was demonstrated by induction of widespread cerebral *β*-amyloidosis following intraperitoneal injections of A*β* rich transgenic brain homogenate into APP-Tg mice [41]. Recently, *in vivo* A*β* propagation was traced using increased GFAP-Luc bioluminescence as an indication of spreading pathology [49]. APP-Tg intracerebral injection of A*β* aggregates purified from APP Tg brain or composed of synthetic A*β* induced widespread A*β* amyloidosis. 

 Prion-like mechanisms within AD are not limited to *β*-amyloid. Work performed by Kfoury et al. demonstrated that aggregates of tau are taken up into cultured cells to initiate misfolding of cellular tau [50]. Further, brain extracts containing misfolded tau, when injected into the brains of tau-transgenic mice, act as seeds to promote further tau misfolding and subsequent spread from the site of injection into neighboring tissues [53]. This pattern of transmission of tau from the point of injection throughout the brain mirrors that of *β*-amyloid [49].

## 7. Prion-Like Mechanisms in Parkinson's Disease

A defining feature of Parkinson's Disease is the appearance of Lewy body inclusions within the brain [86]. These aggregates are primarily composed of the protein *α*-syn. A fragment *α*-syn, the nonamyloid component (NAC), is also observed in AD plaques [87], highlighting the potential for this protein to undergo pathological aggregate formation. There is strong evidence linking *α*-syn to PD. Familial forms of PD often reflect mutations to the *α*-syn gene and wt *α*-syn, when overexpressed, can result in PD-like toxicity [88]. Propagation of aggregates of *α*-syn has been observed in cultured human neurons, initiating formation of Lewy body—like aggregates in a cell-to-cell fashion [59, 61]. This effect was further demonstrated *in vivo* where CNS injection of recombinant *α*-syn seeds, or brain homogenate from mice exhibiting *α*-syn pathology, resulted in progressive induction and dissemination of endogenous *α*-syn aggregation, selective loss of dopaminergic neurons, and progressive deterioration of motor function [62, 66]. Such mechanisms appear to have real-world consequences. The development of *α*-syn deposits in fetal cells transplanted into the brains of Parkinson's patients supports self-propagation of *α*-syn [63, 64, 89]. The postnatal time period for formation of Lewy bodies in the grafted tissues was far less than that typically observed in “natural” PD [90]. This phenomenon was further examined in mouse models recapitulating host to graft pathogenic *α*-syn cell-cell transfer and seeding aggregation [61]. The appearance of protofibrillary deposits within these normal, healthy transplanted cells, in as early as four years, is consistent with a prion-like mechanism of transmission of *α*-syn aggregation. While appearance of Lewy body formation in grafted neurons has often been interpreted as evidence of a prion-like mechanism, an alternate hypothesis is that the host condition provides an environment that is not specific to a prion-like mechanism, which promotes misfolding. 

## 8. Prion-Like Mechanisms in ALS

 Amyotrophic lateral sclerosis is the most common motor neuron disease [89]. Characterized by adult-onset and progressive degeneration of motor neurons, ALS results in paralysis and death within 1–5 years of onset [91]. A proportion of ALS cases are familial (10%), and the remaining are sporadic (90%), yet the clinical manifestations of both forms exhibit a high degree of similarity [92]. Pathological hallmarks of ALS include the misfolded protein inclusions of SOD1 and TDP-43 in motor neurons. Several studies indicate that the misfolding and aggregation mechanism of these proteins likely involves prion-like propagation. These observations indicate that immunotherapeutic targeting of ALS-associated misfolded proteins may be a viable therapeutic strategy. Further, it may provide explanation for the clinically observed spread of atrophy from the focal point of symptom initiation.

A subset of familial ALS has been attributed to mutations in Cu/Zn superoxide dismutase 1 (SOD1), a highly conserved, ubiquitously expressed enzyme responsible for neutralizing superoxide radicals [93]. All observed disease-associated mutations result in a destabilization of the unusually stable SOD1 structure, although to varying degrees, resulting in an increased propensity to misfold [94]. There are indications of the ability of SOD1 to exhibit seeded aggregation and cell-to-cell transmission. Chia et al. demonstrated that misfolded and aggregated forms of SOD1, as either recombinant protein or from mutant SOD1 transgenic spinal cord tissue homogenates, act as amyloid seeds that accelerate formation of new SOD1 fibrils [71]. Subsequently, Grad et al. demonstrated that expression of familial ALS SOD1 mutations (G127X and G85R) in human mesenchymal and neural cell lines induced the misfolding of wild-type, natively structured SOD1. Reminiscent of the species and strain barriers that have been characterized for prion transmission, expression of these SOD1 mutants in mouse cell lines did not induce the misfolding of murine wtSOD1. In addition, it was shown that misfolded wtSOD1 can induce the misfolding of cell-endogenous wtSOD1. Finally, it was demonstrated that aggregated recombinant G127X induced misfolding of recombinant human wtSOD1 in a cell-free system. These observations establish that misfolded SOD1 induces misfolding of natively structured wtSOD1 in a physiological intracellular environment in a manner supportive of direct protein-protein interaction [72]. Münch et al. reported that aggregates composed of the normally folded mutant SOD1 are taken up in tissue culture where they induce misfolding of the soluble mutant protein. These misfolding events were transmissible from cell-to-cell, suggesting the disease self-propagates within the afflicted/infected individual in a manner that bears considerable similarity to the progression mechanisms of prion diseases [73]. 

 TDP-43 is an RNA/DNA binding protein involved in various aspects of RNA metabolism [95]. TDP-43 has been implicated in ALS pathology due to its frequent occurrence in inclusions of sporadic ALS cases [96, 97], as well as the association between dominantly inherited mutations in TDP-43 and familial disease [98–100]. TDP-43 misfolding has also been identified in other proteinopathies including AD [101], frontotemporal degeneration [97], and Lewy body diseases [102]. The mechanism of TDP-43 toxicity in ALS is debated and there is substantial evidence for both gain and loss of function hypotheses [95]. The gain of function hypothesis describes TDP-43 toxicity as a consequence of fragmentation and abnormal localization in the cytosol proceeded by aggregation and inclusion formation [103]. A recent study demonstrated that this toxic aggregation takes on a prion-like seeding mechanism, whereby transduction of HEK293T cells overexpressing TDP-43 with recombinant TDP-43 fibrils triggers fibrillation of the soluble endogenous TDP-43 [78]. Specifically, cell exposure to aggregate seeds induced migration of nuclear TDP-43 into the cytoplasm where it co-localized with the seeding fibrils forming inclusions, characteristic of patient-derived tissue [104]. These inclusions, generated by prion-like nucleated polymerization, were characteristic of patient-derived pathological inclusions in terms of sarkosyl insolubility and ubiquitination. Notably, this prion-like nucleated polymerization reaction may also contribute to loss of function toxic mechanisms through accelerated sequestration of functional TDP-43. It remains to be determined whether this phenomenon can be transmitted between cells, and as such, the implications of TDP-43 prion propagation on pathology and therapeutic interventions remain unclear at this time.

## 9. Prion-Like Mechanisms in Huntington's Disease

Huntington's disease is a genetic disease associated with the cytotoxic misfolding and aggregation of Huntingtin protein as a consequence of variable expansions within a polyglutamine repeat [105, 106]. The direct correlation between the extent of polyQ expansion with propensity for aggregate formation, disease severity, and age of onset strongly implicates aggregated mutant Huntingtin as the causative agent of disease [78, 106, 107]. Several observations point to the ability of mutant Huntingtin to exhibit prion-like propagation as a component of its pathogenic mechanism, including prion-like aggregate morphology [107], conformational diversity [108], cellular uptake of aggregates [78, 80], and a seeding nucleation mechanism of propagation [80]. Specifically, large aggregates of pathogenic polyQ expansion peptides are taken up into cultured cells where they effectively recruit soluble nonpathogenic expansions of polyQ into the aggregate core [80]. These misfolding events persist for several generations following the initial, limited exposure of the cells to extracellular polyQ aggregates. As the release of polyQ aggregates from cells has yet to be demonstrated, the physiological importance of a prion-like mechanism within HD has yet to be established. Nevertheless, this mechanism for progression and amplification of misfolded Huntingtin may have implications for the design of effective therapies for this untreatable disease. Other polyglutamine diseases, such as the spinocerebellar ataxias, may also share a prion-like mechanism [109].

## 10. Prion-Like Propagation as a Universal Basis of Proteinopathies

While the evidence for a prion-like mechanism in a number of neurodegenerative diseases is certainly compelling, it is important that this hypothesis is considered within an appreciation of the biological complexity of these diseases as well as the uncertainties associated with prion biology. There is strong evidence for the ability of the proteins associated with these diseases to self-propagate within biological contexts; the extent to which these events contribute to the progression and pathology of each disease has yet to be determined [110]. While the existence of a common mechanism within these critical diseases is certainly appealing and warrants careful consideration, this should not be to the exclusion of other potential disease mechanisms. For example, the progression and pathologies of N-terminal fragments of tau, which are unlikely candidates for aggregate formation, are consistent with a receptor-, rather than prion-like, mediated mechanism of transmission [111–116]. More generally, other characteristics of the aggregating proteins of the neurodegenerative diseases, including induction of endosome-lysosome defects, may offer alternate mechanisms of disease progression and pathology [117].

## 11. Immunotherapy of Proteinopathies

There have been extensive efforts towards the development of vaccines for the neurodegenerative diseases discussed thus far. These approaches have considered a spectrum of epitopes, as well as a number of strategies for vaccine formulation and delivery. These efforts are extensively reviewed elsewhere: TSEs [118, 119], AD [120], PD [121], ALS [122], and HD [123]. While it is not yet possible to celebrate the development of a successful vaccine for any of these diseases, the preliminary results provide critical proof-of-principle evidence that vaccine-based therapies are possible. It may be appropriate to reconsider the results of these trials, as well as consideration of future vaccine development efforts, from the perspective of a prion-like mechanism of propagation. Specifically, considering the misfolded species of the causative protein as infectious agents that if neutralized through antibody binding may delay or eliminate disease progression. 

## 12. Implications of a Prion-Like Mechanism for Immunotherapy of Neurodegenerative Diseases

**Figure 2:**
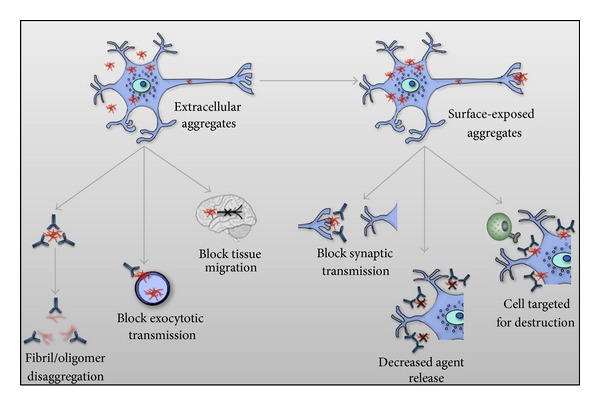
Potential effector functions of immunotherapeutic antibodies in proteinopathies. Misfolded protein-specific antibody responses could function in a neutralizing fashion to bind and block extracellular misfolded protein from spreading to adjacent cells and tissues. These antibodies could also act upon misfolded proteins still associated with diseased cells, thereby decreasing local cell-cell spread, disallowing release of further misfolded protein, and marking cells for destruction by antibody dependent cell mediated cytotoxicity or complement activation.

Consideration of AD, PD, ALS, and HD from the perspective of having prion-like characteristics has immediate implications for therapeutic strategies. For example, the demonstration that transplanted fetal cells will succumb to “infection” by *α*-syn aggregates complicates stem cell therapies to treat diseases like PD [63, 64]. More optimistically, if self-propagation and cell-to-cell transmission represent essential components of disease progression and persistence, this may present an opportunity to use antibodies, or other molecules, for therapeutic benefit. There are a number of ways an antibody could be therapeutic: promoting breakdown of the aggregate, blocking its ability to function as a nucleation seed, or blocking its ability to enter into healthy neighboring cells could all have a positive impact on controlling disease progression (Figure 2).

 A central tenet of this approach is providing the immune system opportunity and access to the misfolding agent. The proteinaceous aggregates, characteristic of these neurodegenerative diseases, exist either as extracellular amyloid plaques or intracellular inclusions (Table 1). However, the localization of toxic pathogenic protein isoforms is much more complex. The rapid emergence of immunotherapeutic strategies for intracellular-based protein misfolding diseases stems from numerous reports identifying a significant extracellular component to the gain-of-function misfolded protein pathogenic mechanism. The misfolded proteins that form intracellular aggregates can be released from cells and may contribute to pathology both through cell-cell transmission and propagation of misfolding, as well as microglial activation and generation of a neurotoxic proinflammatory response. For the prion diseases, conversion of PrP^C^ to PrP^Sc^ occurs at, or near, the cell surface [124, 125]. As such, there is theoretical opportunity for antibodies to limit the interaction required for disease progression. There may be similar opportunity among the other prion-like diseases. For example, PD is often associated with mutations within *α*-syn [126]. While *α*-syn and its aggregates are typically associated with intracellular localization in presynaptic terminal, under pathological conditions the oligomers and protofibrils of *α*-syn have been observed on the plasma membrane [127–129]. Not surprisingly, this surface exposure of the aggregates enables cell-to-cell transmission [130]. This mechanism bears close resemblance to prion transmission and may offer similar opportunity for immunotherapy. Likewise, while SOD1 is normally intracellular the misfolded species is released from the cell, likely promoting disease progression but also offering opportunity for therapeutic intervention [70].

 Importantly, it is not necessary for the oligomers and aggregates to exit the cell to provide targets for immunotherapy. Intrabodies, intracellularly expressed antibody fragments of the antigen-binding domains, offer the means to target intracellular proteins. Intrabodies retain the potential of the immune system to recognize targets of diverse sequence and conformation. An array of intrabodies have been developed and applied towards different protein species associated with the proteinopathies [131–136].

## 13. Challenges to Immunotherapy of Neurodegenerative Proteinopathies

Immunotherapy for proteinopathies is complicated by tolerance of the immune system to antigens of self-molecules. Specifically, T and B cells, which have receptors specific to elements of self-proteins, are deleted or prevented from initiating immune responses [137]. Overcoming immunological tolerance to these disease-associated self-proteins remains a central challenge to vaccine development for neurodegenerative diseases [138]. This is further complicated by the requirement for antibodies to cross the blood-brain barrier to gain access to the misfolded species. Efforts by our group in the development of a prion vaccine demonstrated that the epitope-specific antibodies were present in the CNS at levels approximately three orders of magnitude lower than those in the serum [139]. Others have reported similar ratios of peripheral to central antibodies [140]. In previous active immunization studies of ALS, survival was directly correlated with antibody titres against the misfolded isoform and the poor immunogenicity of the immunizing antigen was indicated as a key limitation of the therapeutic effect [141, 142]. As such, in an effort to increase the amount of antibody that has the potential to induce a therapeutic effect in the CNS, there is a priority to maximize immune responses through epitope selection as well as vaccine formulation and delivery. 

Therapeutic approaches based on the induction of immune responses to self-proteins are overshadowed by potential pathological consequences that may result from the presence of autoreactive antibodies to a normal self-protein. It is important to remember that each of the proteins associated with the proteinopathies is serving dual purpose, fulfilling an important role in its properly folded conformation while exerting pathological consequences in its misfolded state. Immunotherapeutic approaches that fail to acknowledge these conformation-dependent functional differences have potential for deleterious consequences. Conformation-specific immunotherapy greatly reduces the risk of targeting a self-protein as only the misfolded conformations will be targeted. These considerations have been at the forefront of efforts of our lab to develop a prion vaccine. While there are limited phenotypic consequences associated with deletion of the PrP^C^ protein in transgenic animals, suggesting limited consequence to loss of PrP^C^ function, there is potential for gain-of-function alterations as a result of antibody binding. Notably, it has been demonstrated that PrP^C^ binding antibodies in the brain resulted in extensive apoptosis of neurons in the hippocampal and cerebellar regions [143]. Further, high titre, systemic autoreactive antibodies to PrP^C^ may impair the natural function of PrP^C^, resulting in inappropriate cell signal activation or stimulation of suppressor T-cell lymphocytes [144]. 

 Such consequences are not unique to the prion diseases. Most infamously, a clinical trial of AN1792, utilizing an A*β* peptide vaccine to induce immune responses to A*β* aggregates, was halted due to aseptic meningoencephalitis and leukoencephalopathy in a number of the vaccinated patients, emphasizing the importance of antigen and adjuvant selection [145]. Similar cautionary tales have emerged from vaccine development efforts focused on tau where certain immunogens are associated with pathological consequences [146]. Further, development of antibodies specific to cytotoxic oligomeric aggregates and attempts at translation of this therapeutic approach across the proteinopathies, disregarding disease-related specificity, has had conflicting results. Although some antibodies have broad reactivity with relevant oligomeric species and consistent inhibition of cytotoxicity [147, 148], other investigations have demonstrated differential effects on cytotoxicity among the proteinopathies [149], further emphasizing the importance of epitope selection.

 Although most of this discussion will focus on antibody-mediated immunotherapeutic strategies, cell-mediated immune responses have significant implications for disease progression, and thus, the success of immunotherapeutic interventions. An important balance exists in the CNS between neuroprotective responses and injurious proinflammatory responses that is regulated by the interplay between resident microglia and infiltrating T lymphocytes [150]. A transition in the CNS cytokine environment from a protective anti-inflammatory Th2 bias to a proinflammatory Th1 bias and the generated toxic response is implicated in disease progression. In the protein misfolding neurodegenerative diseases, the mechanism of microglial activation and subsequent pathological consequences have yet to be clearly defined but consistently appear to be exacerbated by the presence of misfolded and/or aggregated disease-causing proteins [151–153]. Due to this common component of neuropathology, immunotherapeutic strategies that modulate the T-cell response have been developed. Stimulation of a protective T-cell response through injections of copolymer-1 resulted in prolonged lifespan and improved motor activity in mice expressing mutant SOD1 [154]. In a similar fashion, adoptive transfer of copolymer-1 immune cells results in neuroprotection in a mouse model for Parkinson's disease [155]. In a more disease-specific manner, Iken et al. demonstrated that adoptive transfer of prion specific, Th2 polarized T-cells inhibited prion replication, and prolonged survival in mice challenged with scrapie [156]. The effect of antigen selection on T-cell responses was demonstrated for *α*-syn, where immunization with nitrated *α*-syn polarized CD4+ T-cells to a Th1 phenotype. Adoptive transfer of these T-cells into a PD model enhanced neuronal loss whereas conversion to a Th2 bias in culture prior to adoptive transfer reversed this effect [157]. Cotransfer of vasoactive intestinal peptide (VIP), known to elicit neuroprotective regulatory T-cell responses, with nitrated *α*-syn, reduced microglial activation and neuronal death [157]. Thus, when pursuing active immunization strategies for neutralization of disease-causing conformers, formulations must stimulate conformation-specific antibody responses in the context of neuroprotective T-cell responses. 

## 14. Disease-Specific Immunotherapy for the Proteinopathies

Given the potential consequences associated with induction of immune responses that include reactivity with the natively folded proteins, there is considerable appeal for conformation-specific immunotherapy. The appreciation of prion-like characteristics in other neurodegenerative diseases, in which the misfolded form of the protein is regarded as an infectious agent to be neutralized, strengthens the rationale of targeting the misfolded species. The ability to target disease-associated conformations depends on identification of protein regions specifically exposed upon misfolding. Such disease-specific epitopes (DSEs) offer highly attractive targets for vaccine development. While this approach is conceptually very appealing, identification of DSEs can be problematic, in particular as many of these misfolded proteins aggregate into complexes unsuitable for structural investigations. Fortunately, for each of the diseases discussed thus far, a number of disease-, or conformation-, specific epitopes have been identified.

## 15. Prion Disease-Specific Epitopes

Investigation of the refolding of PrP^C^ into PrP^Sc^ revealed a YYR-motif that is specifically surface exposed in the PrP^Sc^ conformation. Antisera to this epitope immunoprecipitated PrP^Sc^ from infected brain but not PrP^C^ from uninfected brains [158]. However, despite an aggressive vaccination protocol, the immune response was limited to IgM antibodies [158]. The limited immunogenicity of this epitope likely reflects the length of the peptide (three amino acids) as well as immunological tolerance. Using the YYR epitope as a starting point, our lab, through dual optimization of the epitope sequence and length, as well as strategies of formulation and delivery, translated the YYR epitope into a vaccine that induces robust PrP^Sc^-specific IgG antibody responses [139]. Epitope optimization, coupled with fusion of the peptide epitope to a highly immunogenic carrier containing several Th-cell epitopes, circumvented established mechanisms of self-tolerance and facilitated efficient IgM to IgG class switching. This investigation highlights the potential to translate DSEs into functional vaccines. This also indicates that, while identification of DSEs represents a critical first step, additional efforts are often required to translate these targets into vaccines.

## 16. Disease-Specific Epitopes for Alzheimer's

The brains of AD patients are characteristically populated with plaques composed of A*β* peptide as well as neurofibrillary tangles of hyperphosphorylated tau [32]. The A*β* peptide is generated from proteolytic processing of the amyloid precursor protein (APP) via the dual actions of *β*- and *γ*-secretase [159, 160]. The A*β* released from APP exists as either a 40 or 42 amino acid peptide [161]. Of these, the A*β*42 fragment has the greater propensity for aggregation and toxicity [162]. Processing of APP through *α*- and *γ*-secretase results in fragment P3 that is generally regarded as non-toxic, although has been shown to induce apoptosis in neuronal cells [163]. Hyperphosphorylation of tau decreases its affinity for microtubule proteins and facilitates tau misfolding and self-assembly into *β*-sheet rich filaments [164]. Both A*β* and p-tau represent potential targets for disease-specific immunotherapy. 

 The first immunotherapeutic strategies for AD targeted A*β*, due to the link between APP mutation and familial AD, coupled with the predominance of A*β*42 in amyloid plaques [165, 166]. Parenteral and mucosal active immunization with A*β*42 peptide, or passive immunization with A*β*42 monoclonal antibodies, substantially reduced neuritic plaque formation, reactive astrogliosis, and cognitive impairment in transgenic mice [167–171]. Translation of these therapeutic approaches to human patients resulted in drastically different results, leading to the early termination of the AN1792 phase II clinical study. In this incomplete trial, there were no significant differences between treated and placebo groups regarding cognitive testing, and 6% of treated subjects developed encephalitis [172]. Notably, the observed meningoencephalopathy was not linked to A*β*42 antibody titres, and adverse effects were attributed to T-cell and microglial activation [172]. Despite termination of these clinical trials, A*β* targeting immunotherapy has shown great promise, although epitope selection, as well as vaccine formulation and delivery, remains to be optimized. 

 Recent strategies of conformation-specific immunotherapy for AD focus on targeting toxic soluble oligomeric species of A*β*42, as monomeric species and fibrils are deemed nontoxic [162, 173]. Currently, it is unknown which isoform represents the causative agent, as several oligomeric structures with possible disease causing toxic properties have been identified [174–183]. Generating conformation-specific antibodies with a high degree of specificity for oligomeric species, while remaining nonreactive against monomers and nontoxic fibrils, has proven to be challenging. Several studies utilized oligomeric A*β* based vaccines, an improvement on preliminary AD immunotherapeutic strategies involving immunization with A*β*42 peptide. Passive administration of these antibodies resulted in improvements in spatial learning and memory but no effect on clearing A*β* pathology [184]. Although these antibodies preferentially bind higher-order structures, they remain somewhat reactive with monomers and nontoxic fibrillar structures, and this lack of specificity may lead to adverse effects. Antibody crossreactivity with nontoxic conformers following immunization with full length A*β* oligomers was attributed to consistent exposure of the disordered N-terminal segment [185]. Immunization with N-terminally truncated A*β* oligomers successfully generated oligomer conformation specific antibodies reactive with an epitope confined to a loop in residues 20–30 [185, 186]. These antibodies, administered through passive immunization or generated by active immunization, improved cognitive function and facilitated maintenance of synaptic plasticity in early stages of disease, prior to plaque formation. Importantly, this study demonstrated that neutralization of oligomeric A*β* species by conformation-specific antibodies was sufficient to ameliorate neuropathology in transgenic disease models. 

Interestingly, conformation selective endogenous antibodies, reactive against oligomeric A*β*, have been identified in serum and CSF [187]. These autoantibodies exhibited increased reactivity against pathogenic oligomeric and/or posttranslationally modified A*β* species and were less abundant in patients with advanced AD compared to age-matched controls [188, 189]. Thus, passive administration of intravenous IgG (IVIgG) was proposed as a potential AD therapy. These polyclonal antibodies were capable of inhibiting A*β* oligomerization, reducing A*β* oligomer toxicity in cell culture, and ameliorated cognitive deficits in APP/L transgenic mice [189–192]. The use of IVIgG therapy was already FDA approved accelerating the translation of this therapy into AD clinical trials [193]. Although these antibodies possess promising therapeutic potential, human clinical trials have yet to demonstrate consistent therapeutic effects. Preliminary studies demonstrated a reduction in CSF A*β* coupled with an increase in serum A*β*, and an inhibition of cognitive decline during treatment [193, 194]. In a recent study performed by Dodel et al. IVIgG therapy did not reiterate previous observations and a significant alteration of AD biomarkers or amelioration of symptomatic effects was not observed [195]. Although IVIgG therapy is quite promising, in order to conclusively assess the therapeutic potential of IVIgG therapy for AD, further studies must be performed with larger sample sizes and longer IVIgG treatments [195].

 Building on investigations of A*β* targeted therapies, conformation-specific targeting of hyperphosphorylated tau is currently being pursued. Although the characteristic tau aggregates are interneuronal, demonstrated neuronal uptake of antibodies and cell-cell transmission of tau misfolding further strengthen the feasibility of this approach [43, 196]. Active immunization with a phosphorylated tau peptide epitope or passive immunization with phosphotau-specific antibodies reduced tau aggregate pathology and delayed functional impairments in an aggressive transgenic model for Frontotemporal Dementia [197, 198]; however, the therapeutic effects declined with disease progression [197]. These preliminary results suggest conformation-specific targeting of tau is also a promising therapy for AD, which could be used in conjunction with A*β*-targeted therapies.

 In addition to antibody neutralization of toxic A*β* and tau conformers, a novel application of immunotherapy for AD involves selection of antibodies or intrabodies that either promote the formation of P3 (to the exclusion of A*β* fragments) or facilitate the sequestering and degradation of A*β*. In one such approach, screens conducted to identify intrabodies that possess *α*-secretase-like activity identified a number of promising molecules. The intrabody (iAB) c23.5 possesses serine protease-like activity and cleaved the A*β* fragment into nontoxic fragments [199]. Another intrabody, hk14, with carboxypeptidase-like activity, was able to trim the A*β*42 peptide into its less toxic A*β*40 counterpart [200]. Intrabody, sFv*β*1, promotes *α*-secretase processing of APP and, when fused to an endoplasmic reticulum retention signal, traps APP in the ER and promotes its degradation to achieve a dramatic reduction in A*β* production [201]. Intrabody H1v2 recognizes the central region of A*β* to reduce aggregation and cellular toxicity [201]. Such intrabodies have the potential to be delivered through adeno-associated virus (AAV) to reduce plaque formation *in vivo* [202, 203].

## 17. Disease-Specific Epitopes for Parkinson's

The oligomeric isoform of *α*-synuclein is a possible causative agent of PD. Elimination of this pathogenic protein isoform has the potential to modify the course of disease [204]. Initially, immunotherapeutic targeting of *α*-syn was based on the premise that pathogenic oligomeric isoforms relocate from the cytosol to the plasma membrane, where they are accessible to circulating antibodies [121, 128, 205]. The recent discovery that toxic *α*-syn isoforms can be secreted and propagate aggregation cell-to-cell through a prion-like mechanism has strengthened the rationale for PD immunotherapy [61, 88]. Several vaccination strategies for targeting/neutralizing toxic *α*-syn oligomers have been examined including active and passive immunization and delivery of intrabodies. 

Active immunization with full-length *α*-syn achieves a reduction in the pathogenic, membrane-associated *α*-syn aggregates in a manner that correlates with antibody-titre [206]. Characterization of these antibodies identified reactivity with several C-terminal epitopes of *α*-syn. With passive immunization, antibodies against these epitopes entered the CNS, cleared *α*-syn aggregates, and ameliorated neurological symptoms in a mouse model for Lewy Body Disease [207]. In both studies, the authors concluded that reduction of aggregates was due to antibody binding of membrane-associated oligomers, internalization of the complex, and lysosomal activation. Recently, AFFiRiS produced a vaccine consisting of a short peptide mimetic of the *α*-syn sequence/structure, fused to an immunogenic carrier, and formulated in Alum [208]. The antigen was designed to stimulate strong B-cell responses, in the absence of damaging T-cell responses, through optimization of the peptide mimetic length. This vaccine has a stronger safety profile through enhanced specificity for *α*-syn, with no crossreactivity with *β*-syn, which has neuroprotective properties through prevention of *α*-syn aggregation and oxidation [209]. These vaccines demonstrated a reduction in cerebral *α*-syn and amelioration of neurological symptoms associated with *α*-syn toxicity in transgenic models of disease and are currently being tested in Phase I clinical trials. 

In these investigations, the generated/administered antibodies exhibited a high affinity for the toxic *α*-syn oligomers that coincided with reactivity with nonpathogenic isoforms. Specific reactivity with pathogenic isoforms was aided by the selective membrane association and exposure of these conformations. However, the soluble *α*-syn monomers are not shielded from antibody binding by their primary localization in the cytosol, as exogenously administered monoclonals can be internalized where they inhibit aggregation of intracellular *α*-syn oligomers [210]. In addition, oligomers and monomers of *α*-syn have been detected in CSF, blood plasma, and interstitial fluid in the brain [211–213]. As such, the physiological and pathological roles of *α*-syn, and subsequently the consequences of its extracellular depletion, have yet to be fully elucidated. Based on these observations, conformation-specific targeting of toxic *α*-syn isoforms remains the most viable strategy.

 Similar to the results observed with active and passive immunization, intrabodies reactive with monomeric *α*-syn prevent aggregation and formation of oligomers and protofibrils in cell culture [134, 135] and cell-free models [214] either by stabilizing the monomeric structure [134, 214] or by directly neutralizing regions of the protein that facilitate aggregation [135]. Subsequent investigations focused on the design of conformation-specific intrabodies that exclusively react with pathogenic isoforms of *α*-syn. *α*-syn is a natively unfolded protein, but there are several conformations that *α*-syn can adopt, including oligomers, protofibrils, and large fibrillar structures found in Lewy Bodies [67, 215, 216], although the small oligomer aggregates have the highest toxicity [217]. Emadi et al. generated a single-chain antibody fragment specific for the oligomeric conformation [218]. This scFv, D5, inhibits formation of *α*-syn fibrils *in vitro* and neutralizes extracellular toxicity in neuroblastoma cells when coincubated with oligomeric *α*-syn treatments. Cellular toxicity was further reduced through fusion of D5 to a secretion signal sequence, whereby intracellular intrabody-oligomer complexes were secreted from the cell, eliminating the toxicity of overexpressed *α*-syn in cell culture [219]. Importantly, intrabodies facilitating secretion of all isoforms had only partial effects on toxicity, and intrabody neutralization in the absence of secretion had no effect on toxicity [219]. Notably, conformation-specific intrabodies can differentiate oligomeric states, as demonstrated with D5 and syn-10 H, that recognize dimeric/tetrameric and tetrameric/hexameric oligomers, respectively [220]. The ability to specifically neutralize different conformational species during the oligomerization process enables further investigation into the role of each isoform in disease pathology and identification of any differential therapeutic effects resulting from specific isoform neutralization. 

 Although these cell culture investigations are promising, translation of this effect into *in vivo* scenarios has yet to be demonstrated. Considering the potential technical and safety issues with recombinant DNA technology and viral delivery in humans [121], the ability of exogenously applied antibodies to enter cells [210], and the prion-like propagation of *α*-syn [59, 63], perhaps a fusion of the conformational specificity achieved in the intrabody investigations with an active or passive immunization approach, may be the most viable strategy. 

## 18. Disease-Specific Epitopes of ALS

The causative agent of the majority of familial ALS cases is mutated and misfolded SOD1. Although SOD1-linked familial ALS is relatively rare, evidence suggests that wtSOD1 can also misfold, leading to the surface exposure of similar misfolding-specific epitopes, and may be a contributing factor to sporadic ALS, due to the presence of misfolded wtSOD1 in the spinal cord of sporadic ALS patients [221–225]. The role of misfolded SOD1 in sALS is still an area of debate, as some studies report an absence of misfolded SOD1-specific antibody reactivity with wtSOD1 in spinal cord tissue of sALS patients [226, 227]. This does not, however, rule out the involvement of misfolded wtSOD1 in sALS pathology but may indicate a difference in the structural destabilization of SOD1 in familial versus sporadic ALS. Nonetheless, the clinical manifestations of sporadic and familial ALS exhibit a high degree of similarity, indicating the potential for application of therapies that are effective in familial ALS to at least a portion of sporadic ALS cases [92]. This discussion will focus on SOD1 immunotherapy as the emergence of TDP-43 as a key player in sporadic ALS is relatively recent, and immunotherapeutic strategies targeting this protein have yet to be demonstrated.

Immunotherapeutic strategies targeting SOD1 have involved both active and passive immunization. Active immunizations were performed with either recombinant mutant or WT apo-SOD1, as metal depletion induces misfolding, and, in turn, the surface exposure of epitopes concealed in the native structure [141, 228]. These strategies delayed disease onset and extended the lifespan of G37R [141] or low copy number G93A transgenic mice [228]. Further, passive immunization involving intraventricular infusion of mutant SOD1 specific antisera significantly delayed disease and prolonged lifespan in the aggressive G93A disease model [141]. Misfolded SOD1 was successful in inducing misfolded SOD1-specific antibodies, but the observed therapeutic effect occurred in conjunction with wtSOD1 reactivity. Consequently, a conformation-specific immunotherapeutic, capable of neutralizing or reducing the toxicity of misfolded SOD1, without interfering with the protective function of nonpathogenic SOD1, is the ideal strategy. 

The feasibility of conformation-specific targeting of misfolded SOD1 was established with the development of the surface exposed dimer interface (SEDI) antibody [229]. The SEDI antibody binds an epitope within the hydrophobic dimer interface that is selectively exposed following either mutation- or oxidation- induced destabilization of the SOD1 dimeric structure. Application of the SEDI antibody established the presence of misfolded SOD1 aggregates in ALS mouse models of disease and in spinal cord tissues of familial ALS patients [226, 229]. The development of a conformation-specific immunotherapy for familial ALS is complicated by the high degree of patient-based variation in SOD1 mutations. The SEDI antibody reacts with the dimer interface of a variety of SOD1 mutants, enabling broad application to cases of ALS induced by SOD1. Following this initial investigation, additional misfolded SOD1-specific antibodies were developed that bind conserved regions of disorder in misfolded SOD1: USOD, specific for the unfolded regions of the *β*-barrel [227], DSE2 [69, 72], which recognizes the disordered electrostatic loop, and DSE1a [72], a modified version of SEDI with improved specificity for misfolded monomeric SOD1 via reactivity against an irreversibly oxidized cysteine residue. These investigations provide proof-of-principle evidence for targeting the pathogenic isoform of SOD1 and their potential translation into immunotherapeutic strategies.

 The first conformation-specific immunotherapy efficacy study for ALS involved passive immunization of SOD1^G93A^ mice with misfolded SOD1-specific monoclonal antibodies or their binding fragments [230]. This decreased mutant SOD1 levels and increased survival, with no adverse effects in mice expressing wild-type SOD1. The induction of antibody responses with high specificity for monomeric SOD1 through active immunization was recently demonstrated with the translation of the SEDI antibody epitope into a multiple antigenic peptide vaccine [142]. This vaccine demonstrated a therapeutic effect with delays in symptom onset and disease progression, as well as an increase in survival in the less aggressive G37R disease model. However, in the aggressive G93A disease model, there was no significant effect on disease progression or survival, but a significant delay in symptom onset was observed. 

## 19. Disease-Specific Epitopes of Huntington's

The expanded polyQ tract in pathogenic mutant Huntingtin protein (mHtt) generates a misfolded conformation that undergoes proteolytic cleavage to produce N-terminal fragments with a high propensity for aggregation [231, 232]. These intracellular fragments are problematic for traditional immunotherapy approaches. The aggregation prone N-terminal fragment contains four regions that have been targeted for disease-specific intrabody development. These conformation-specific intrabodies exhibit preferential binding of the toxic N-terminal fragments and are nonreactive with the primarily full-length wild-type protein, which is essential as reduction of wild-type protein amplifies the effect of mHtt aggregates [233]. 

Although the expanded polyQ tract is the site of disease-inducing mutation, and an obvious first choice for disease-specific therapy, intrabody binding to this site has been shown to exacerbate cytotoxicity in cell culture and organotypic brain slice models [234, 235], as was similarly observed with a subset of antibodies with nonspecific reactivity towards aggregated oligomers [149]. Subsequent work focused on regions of the exon1 translational product adjacent to the polyQ tract: the short N-terminal region preceding polyQ, and the proline-rich domain and short C-terminal segment downstream of polyQ. 

The first 17 amino acids of mHtt potentiates toxicity of the oligomeric fragments through regulation of several components of mHtt-induced pathology, including subcellular trafficking between the nucleus and cytosol, fragment aggregation, and degradation [236–239]. The proline-rich domain is also a determinant of fragment aggregation and is implicated in aberrant protein-protein interactions that contribute to HD pathology [240, 241]. The C-terminal domain modulates cellular toxicity of the N-terminal fragment, but its function remains unclear [123]. Intrabodies designed to each of these regions are all capable of preventing aggregation and neutralizing the cytotoxic properties of mHtt fragments in cell culture [234, 236, 240, 242–244]. Potential mechanisms explaining these effects include stabilization of a nontoxic conformation of mHtt, acceleration of mHtt turnover through enhanced degradation, and inhibition of aberrant interactions [240, 243]. 

Translation of this therapeutic approach into *in vivo* disease models has had contradicting results. Several intrabodies have been screened for therapeutic effect in Drosophila and mouse disease models expressing mHtt. Coexpression with intrabodies targeting the N-terminal region of the mHtt fragment demonstrated a reduction of mHtt aggregates and neuronal cell protection in both models. Unfortunately, therapeutic effects were restricted to earlier stages of disease, and mHtt-induced pathology overwhelmed any intrabody effects in older subjects and, in some models, intrabody expression exacerbated disease [232, 245, 246]. Thus far, the most promising intrabody investigations, targeting regions downstream of the polyQ tract, have demonstrated reduction of aggregates and amelioration of neurological symptoms in Drosophila and mouse HD models [244, 246, 247]. 

From these investigations, it is clear that mHtt aggregation propensity and cytotoxicity can be manipulated through intrabody binding. However, care must be taken to ensure that this interaction stabilizes conformations that negate aggregation and toxicity rather than those that potentiate it. Further, due to the critical role of exon 1 in modulating Htt protein function and localization, targets in this region must be carefully selected to avoid interfering with these processes, and thus exacerbating pathology. The differential therapeutic effects observed in HD models indicate a need for optimization of intrabody design and delivery. The indication that mHtt-induced pathology may involve a prion-like mechanism of propagation strengthens the argument that neutralization or inhibition of aggregate formation may be a viable therapy for HD. However, the exclusively intracellular intrabody strategy does not address potential extracellular roles of mHtt in disease pathology and therefore may not be sufficient for complete neuronal protection.

## 20. Conclusions

Vaccines are among the most powerful tools for ensuring human and animal health. Diseases that previously afflicted millions of people and represented critical threats to human health and survival have been rendered historical footnotes through the development and implementation of successful vaccines. Human health is facing a new type of epidemic, an epidemic of aging. As a corollary of increased lifespans enjoyed as a consequence of the advances in medicine there is increased prioritization of diseases associated with aging. This includes a number of neurodegenerative diseases, which through a combination of late onset and/or longer latency periods are affecting a greater proportion of our population. This includes diseases such as Alzheimer's disease, Parkinson's disease, Amyotrophic Lateral Sclerosis, and Huntington's disease. Additionally, while prion diseases of humans represent a relatively minor health concern, they can also represent an aging-associated neurodegenerative disease that shares many mechanistic features of the more prevalent neurodegenerative disorders. More importantly, particular characteristics that used to be uniquely attributed to the prion diseases are now being suggested as common features across this spectrum of neurodegenerative disorders. This paradigm shift may have critical implications of how we approach the treatment and prevention of these diseases. 

 The approach of considering self-antigens associated with pathophysiological states opens a wealth of opportunities. Within these includes the development of vaccines for neurodegenerative diseases, such as AD, and prion diseases such as CJD and CWD. A fascinating common denominator of these diseases (or at least within variants of these diseases) is the occurrence of misfolding of a self-protein into a pathological conformation. This includes PrP^C^ for prion diseases, superoxide dismutase 1 for ALS, *α*-synuclein for PD, *β*-amyloid peptides for AD, and expanded polyQ Huntingtin in HD. These instigating proteins are critical for understanding the mechanisms of disease as well as providing targets for vaccine development, the rationale traditionally being that the induction of antibody or cellular responses against the culprit protein will enable the system to clear the pathological entities associated with these diseases and that clearance of these entities could stop or delay the progression of the disease. It is likely the lessons learned in each of these distinct, yet functionally related, challenges will guide and inform each other. 

## Figures and Tables

**Table 1:** Evidence for prion-like mechanisms in common neurodegenerative disorders.

Disease	Misfolded protein	Aggregate cellular location	Self-propagation	Cell-cell spread	Tissue migration	Transmission	Resistance to degradation
TSEs	Prion	Intracellular [33] Extracellular [34]	Yes	Yes	Yes	Yes	Yes
Alzheimer's	Amyloid beta	Intracellular [35] Extracellular [36]	Yes [37]	Yes [38]	Yes [39–41]	Yes [39, 42, 43]	Yes [44, 45]
Alzheimer's	Tau	Intracellular [46] Extracellular [47, 48]	Yes [49]	Yes [50–52]^,^	Yes [53, 54]	Yes [43, 55]	Yes [56, 57]
Parkinson's	*α*-Synuclein	Intracellular [58] Extracellular [59, 60]	Yes [59]	Yes [59, 61–65]	Yes [62, 66]	Possibly [66]	Yes [67, 68]
ALS	SOD1	Intracellular [69] Extracellular [70]	Yes [71, 72]	Yes [73]	Possibly [74]	No	No ↑ degradation [75, 76]
ALS	TDP-43	Intracellular [77]	Yes [78]	No	No	No	No
Huntington's	Huntingtin	Intracellular [79]	Yes [78, 80, 81]	Possibly [80]	Possibly [82]	No	Yes [83]
